# Wet-Spun Trojan Horse Cell Constructs for Engineering Muscle

**DOI:** 10.3389/fchem.2020.00018

**Published:** 2020-02-20

**Authors:** Anita F. Quigley, Rhys Cornock, Tharun Mysore, Javad Foroughi, Magdalena Kita, Joselito M. Razal, Jeremy Crook, Simon E. Moulton, Gordon G. Wallace, Robert M. I. Kapsa

**Affiliations:** ^1^ARC Centre for Electromaterials Science, Intelligent Polymer Research Institute, University of Wollongong, Fairy Meadow, NSW, Australia; ^2^Centre for Clinical Neurosciences and Neurological Research, St. Vincent's Hospital Melbourne, Fitzroy, VIC, Australia; ^3^School of Engineering, Royal Melbourne Institute of Technology, Melbourne, VIC, Australia; ^4^School of Medicine and Faculty of Health, Deakin University, Waurn Ponds, VIC, Australia; ^5^Institute for Frontier Materials, Deakin University, Waurn Ponds, VIC, Australia; ^6^Illawarra Health and Medical Research Institute, University of Wollongong, Wollongong, NSW, Australia; ^7^Department of Surgery, St Vincent's Hospital, The University of Melbourne, Fitzroy, VIC, Australia; ^8^Department of Biomedical Engineering, Faculty of Science, Engineering and Technology, Swinburne University of Technology, Hawthorn, VIC, Australia

**Keywords:** alginate fibers, myoblasts, wet-spun, muscle engineering, biosynthetic muscle scaffold

## Abstract

Engineering of 3D regenerative skeletal muscle tissue constructs (skMTCs) using hydrogels containing muscle precursor cells (MPCs) is of potential benefit for repairing Volumetric Muscle Loss (VML) arising from trauma (e.g., road/industrial accident, war injury) or for restoration of functional muscle mass in disease (e.g., Muscular Dystrophy, muscle atrophy). Additive Biofabrication (AdBiofab) technologies make possible fabrication of 3D regenerative skMTCs that can be tailored to specific delivery requirements of VML or functional muscle restoration. Whilst 3D printing is useful for printing constructs of many tissue types, the necessity of a balanced compromise between cell type, required construct size and material/fabrication process cyto-compatibility can make the choice of 3D printing a secondary alternative to other biofabrication methods such as wet-spinning. Alternatively, wet-spinning is more amenable to formation of fibers rather than (small) layered 3D-Printed constructs. This study describes the fabrication of biosynthetic alginate fibers containing MPCs and their use for delivery of dystrophin-expressing cells to dystrophic muscle in the *mdx* mouse model of Duchenne Muscular Dystrophy (DMD) compared to poly(DL-lactic-co-glycolic acid) copolymer (PLA:PLGA) topically-seeded with myoblasts. In addition, this study introduces a novel method by which to create 3D layered wet-spun alginate skMTCs for bulk mass delivery of MPCs to VML lesions. As such, this work introduces the concept of “Trojan Horse” Fiber MTCs (TH-fMTCs) and 3d Mesh-MTCs (TH-mMTCs) for delivery of regenerative MPCs to diseased and damaged muscle, respectively.

## Introduction

In absence of effective pharmaceutical treatments for the long-term functional restoration of muscle tissue mass or function lost due to disease or trauma, numerous cell-based strategies have emerged to improve regenerative outcomes in damaged and dysfunctional skeletal muscle (Emery, 2002). These strategies include myoblast transfer therapies, growth factor delivery, stem cell delivery, gene therapy, and combinations of each (Emery, 2002; Mendell et al., 2010; Gombotz and Wee, 2012; Proto and Huard, 2013).

While skeletal muscle is a regenerative tissue by comparison with other soft tissues, regeneration is dependent on the activation of a pool of satellite cells (SCs) from their quiescent state, and in the case of larger traumas or degenerative disease, this process is hindered, and medical intervention is required. The function of the SCs is to initiate (in the response to major injury) cell-based myoregeneration by proliferative differentiation of muscle precursors that eventually fuse into multi-nucleated myotubes, forming functional myofibres whilst retaining a myoregenerative muscle precursor cell (MPC) niche (Proto and Huard, 2013).

In degenerative myopathies such as the muscular dystrophies, regenerative MPC loss occurs more rapidly than in non-dystrophic muscle due to progressive myofibre degeneration and removal from the functional niche. This places excessive demand on the regenerative MPC pool to replace the lost muscle fibers until the MPC pool is exhausted. This regenerative/functional niche dynamic underpins the progressive skeletal muscle weakness and volume loss characteristic to the disease. By example, the X-linked congenital Duchenne's Muscular Dystrophy (DMD), is caused by mutation of the gene encoding the cytoskeletal protein dystrophin results in a lack of functional dystrophin (Mendell et al., 2010), resulting in membrane destabilization and loss of muscle tissue (Blake et al., 2002). This dystrophic process can be ameliorated by implantation of dystrophin-expressing donor cells that fuse with the host muscle and provide functional dystrophin. However, past attempts at Myoblast Transplantation Therapy (MTT) have had mixed success in dystrophic *mdx* mice but failed in humans due to factors including (i) limited migration of the cells from the injection site, (ii) immune rejection of the cells by the host muscle, and (iii) general high-level post-implant cell death. Corrective gene editing (cGE) Strategies have been designed and successfully applied for editing out the dystrophin gene mutation *in vitro* and *in vivo* (Kapsa et al., 2001, 2003; Wong et al., 2005; Bengtsson et al., 2017). These facilitate the possibility of providing autologous myoregenerative cells with corrected dystrophin loci to restore non-dystrophic functional/myoregenerative niche dynamics in dystrophic muscle provided that cells can be effectively delivered to the dystrophic muscle. In the case of volumetric muscle loss (VML) injury, the situation is similarly dependent on cell delivery/matrix technology that allows for myoregenerative activity to be successfully localized to the VML lesion.

To be of clinical benefit, transplanted myoblasts must either fuse with the host myofibres to or form *de novo* skeletal muscle via fusion of the donor cells (Skuk et al., 2004, 2006, 2011). The current “gold-standard” methodology for introduction of regenerative cells to muscle involves Bolus injection. This is of limited efficacy due to failure of donor cells to engraft away from the injection site, as well as immune reaction toward the donor cells (Skuk et al., 2004, 2006, 2011). This technique has been attempted for the delivery of myoblast transfers and stem cell doses to diseased muscle, and in many cases, studies have used alginate as a protecting hydrogel for the donor cells (Borselli et al., 2011). Other studies have examined the use of thermoplastic implantable fiber structures which exploit their linear nature in aiding the alignment of transplanted myoblasts to encourage myogenesis and eliminate difficulties with dystrophin localization (Razal et al., 2009).

Alginate is an abundant, naturally occurring anionic polysaccharide, harvested from marine brown algae or less commonly, from bacterial sources (Augst et al., 2006; Draget and Taylor, 2009; Lee and Mooney, 2012). It is a co-polymer consisting of (1-4)-linked β-D-mannuronate (M) and its C-5 epimer α-L-guluronate (G) residues, covalently linked together in varying sequences or blocks, typically homopolymer blocks and poly-alternating sequence blocks (e.g., GGGG, MMMM, and GMGM). Due to its biocompatibility, low toxicity and almost temperature independent gelation in the presence of divalent cations, alginate has been the subject of extensive investigation in wound healing roles, in the transplantation of cells, and in the delivery of pharmaceuticals and immobilized bioactive agents (Gombotz and Wee, 2012; Lee and Mooney, 2012; Falzarano et al., 2013).

Current high-level complexity possible in material design of medical alginate structures has revived exploration into biofabrication techniques for implantable alginate/cell construct technologies. Several additive assembly techniques such as nanofibrous electrospinning, microbead encapsulation, wet-spinning, casting, and bioplotting or 3D printing have each been studied as a means of encapsulating cells within alginate constructs. Of these processes, wet-spinning is desirable due to its high throughput for fiber production and as the output mimics the linear geometric characteristics of native muscle. Furthermore, wet spinning allows for bio-functional components (including cells) to be deposited, woven or knitted into defined structures with minimal effect to biological function (Chen et al., 2009). In addition as with other additive biofabrication technologies such as 3D printing, wet spinning facilitates the precise placement within and release of bio-factors from fibers, enabling more effective and reproducible fabrication of functionally defined devices and structures (De Ruijter et al., 2006; Mironov et al., 2009). Control of the processing parameters during the fabrication of wet-spun fibers, such as (in the case of alginates) the coagulation or non-solvent bath concentration, the feedstock character and concentration and draw rate (injection speed/collection spindle speed), can be used to tune the throughput, porosity, and consistency of the generated fibers (Qin, 2008; Peng et al., 2012).

Earlier studies by others (Hill et al., 2006; Borselli et al., 2011), confirmed Alginate as being useful for delivery of regenerative cells to muscle injury sites through compliance with base requirements for successful re-engineering of damaged muscle (Lee and Mooney, 2012). However, these studies applied cell-laden alginate structures in “patch” formats covering VML injuries to replace bulk muscle, but did not apply Alginate as an intramuscular implant to restore dystrophin expression to dystrophin-negative muscle.

This study applies Cell-laden Alginate “Trojan Horse” structures implanted into dystrophic *mdx* mouse gastrocnemius muscles to evaluate their potential to remodel dystrophic muscle with dystrophin-expressing cells. In particular, this study presents an optimized alginate/myoblast wet-spinning protocol alternative to 3D bioprinting. This protocol allows high-viability encapsulation of muscle precursor cells (MPCs) within well-characterized alginates as biocompatible fibers to form Trojan Horse fiber muscle tissue constructs (fMTC) that hide the introduced regenerative cells from host immune response. These Trojan Horse fMTCs (TH-fMTCs) containing transgenic wild type (i.e., non-dystrophic) β-Galactosidase-expressing mouse myoblasts encapsulated within alginate fiber carriers, were implanted into dystrophic (*mdx*) mouse muscle (mouse model of DMD). This method of cell encapsulation within an alginate hydrogel was compared to implantation of poly(DL-lactic-co-glycolic acid) copolymer (PLA:PLGA) topically-seeded with myoblasts or cells implanted by themselves as a cell bolus. Applicability of the TH-fMTCs to replacement of bulk muscle for VML injury was then further explored by in-process assembly of TH-fMTCs into 3D Trojan Horse Mesh-MTC (TH-mMTC) scaffolds using an additive wet-spinning approach.

## Materials and Methods

### Materials

A buffer solution of 4-(2-hydroxyethyl)-1-piperazineethanesulfonic acid (HEPES, Biochemical, #L505388) was made up to a concentration of 308 mM in a 20 mM sodium chloride (NaCl) solution. Alginic acid with sodium salt (Sigma, #035K0205) was made to concentrations of 2 and 4% (wt/v) utilizing an appropriate volume of distilled and autoclaved water at 60°C with stirring. Primary myoblasts were grown to 80% confluence in a series of 30 T-75 flasks in 20 ml of Dulbecco's Modified Eagle's Medium (DMEM) at 37°C. The cells were then trypsinised and centrifuged at 280 g for 2 min to pellet the cells. The cells were then resuspended in HEPES solution to concentrations of 20, 40, and 60 million cells/ml. Mixing with corresponding alginate solutions resulted in cell concentrations of 10, 20, and 30 million cells/ml for both 1 and 2% (wt/v) alginate concentration.

### Feedstock (Spinning Dope) Preparation

Alginate feedstocks containing no cells were made for myoblast-loaded feedstocks: A buffer solution of 4-(2-hydroxyethyl)-1-piperazineethanesulfonic acid (HEPES, Biochemical, #L505388) was made up to a concentration of 308 in a 20 mM sodium chloride (NaCl) solution. Alginate solutions were made to concentrations of 2 and 4% (wt/v) utilizing an appropriate volume of deionized and autoclaved water at 60°C with stirring.

Cultured donor C57BL/10ScSn^β−Gal^/SVHM (C57/BL10J^β−Gal^; wild-type-βGal; *wt*-βGal) mouse myoblasts were grown to 80% confluence in 20 ml of Dulbecco's Modified Eagle's Medium (DMEM) at 37°C in a series of 30 T-75 flasks. The cells were then trypsinised and centrifuged at 2,200 rpm for 2 min to pellet the cells. The cells were then resuspended in HEPES solution to concentrations of 20, 40, and 60 million cells/ml. Mixing with corresponding alginate solutions resulted in cell concentrations of 10, 20, and 30 million cells/ml for both 1 and 2% (wt/v) alginate concentrations.

### Donor Myoblast Cell Preparation

Male C57BL/10ScSn^β−Gal^/SVHM mice, 5–6 week of age were obtained (Bioresources Center, St. Vincent's Hospital, Melbourne, Australia), exposed to a 12 h day/night cycle and fed *ad*-*libitum* until they were required for muscle culture. All animal handling aspects of this study were carried out in accordance with the recommendations of the Australian Code of Practice for the Care of Animals for Scientific Purposes (NHMRC) as outlined in protocol SVHM86/06 and approved by the St. Vincent's Hospital Animal Ethics Committee. After cervical dislocation, skeletal muscle was removed from the hind limbs of the mice and primary myoblast cultures prepared as described elsewhere (Todaro et al., 2007).

Briefly, the muscle tissue was finely minced using scissors and digested with 10 mg ml^−1^ Collagenase D (Roche), 2.4 U ml^−1^ Dispase II (Roche), and 2.5 mM CaCl_2_ for 2 h. The muscle slurry was filtered through 70 and 40 μm filters, respectively. Red blood cells were lysed by 5 min, room temperature incubation with 17 mM tris(hydroxymethyl)aminomethane and 144 mM ammonium chloride. The myoblast cultures were preplated once for 20 min, once for 40 min, once for 2 h, and then at 24 h intervals for the next 4 days in proliferation medium consisting of Hams/F10 (TRACE) supplemented with 20% fetal bovine serum (Invitrogen), 2.5 ng ml^−1^ bFGF (PeproTech Asia), 2 mM L-glutamine (Invitrogen), 100 U ml^−1^ of penicillin (Invitrogen), and 100 mg ml^−1^ streptomycin (Invitrogen). Adherent myoblasts at preplate 9 were cultured in proliferation medium at 37°C and 5% CO_2_ and passaged at >80% confluence.

### Wet Spinning

**Figure 1:**
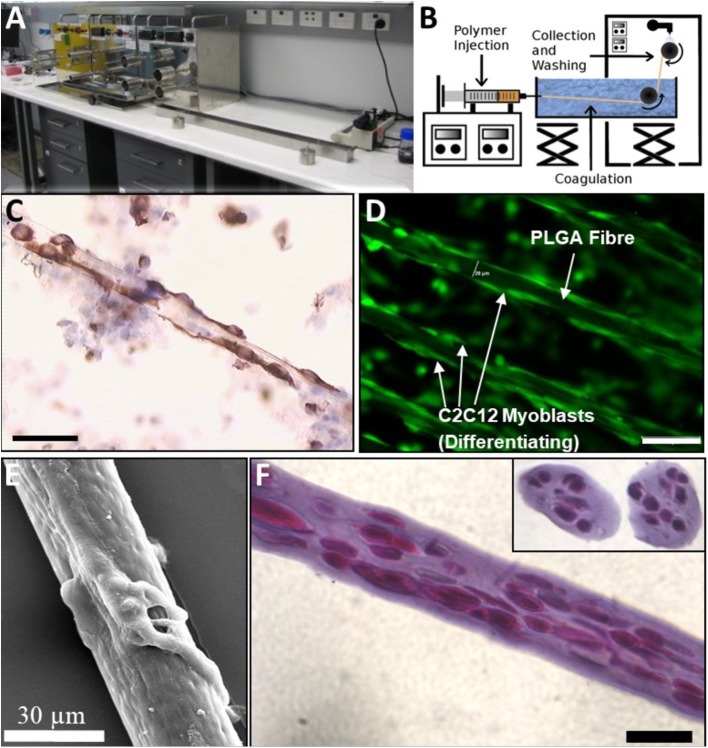
Wet-Spinning BioSynthetic Myoblast Fibers (PLGA and Alginate): Comparison of native Muscle, PLGA and “Trojan Horse” Alginate Fibers. **(A)** Apparatus. **(B)** Line Schematic. **(C)** Native Muscle Fibers and myoblasts immunostained with Desmin: early culture. **(D)** PLGA Fibers with myoblasts and myotubes attached externally (immunostained with Desmin). **(E)** SEM of PLGA fiber with myotubes attached. Myoblasts on the PLGA fibers were seen to spread and differentiate longitudinally without evidence of retained proliferative capacity under differentiation conditions *in vitro*. **(F)** 1% Alg Trojan Horse Fibers with 2 × 10^7^ myoblasts/ml (longitudinal and cross sections inset, Hematoxylin and Eosin). Unless otherwise specified, size calibration markers are 100 μm.

A wet spinning arrangement as shown in Figures 1A,B was used to fabricate cell-impregnated poly(DL-lactic-co-glycolic acid) copolymer (PLA:PLGA) (Figures 1D,E) and alginate (Figure 1F) Trojan Horse fibers. The unit consists of a digitally controlled, four-beam stainless steel spindle, with outermost diameter of 40 mm, and a second controller with a piston for directed fiber output along a single axis.

For wet spinning cell-containing Alginate fibers, an aqueous coagulation bath (L = 1 m) of 2% calcium chloride (Sigma, #48H0106) was used for cross-linking the fibers. Myoblasts were encapsulated in Pronova ultra-pure low viscosity, high guluronate sodium alginate (20–200 mPa.s for 2% w/w solution at 25°C, >60%G, *ca*. 75–200 kDa) obtained from Novamatrix for all *in vitro* and *in vivo* experiments.

Prior to encapsulation, myoblasts were suspended in 2 x cell buffer (300 mM NaCl, 20 mM HEPES) at a concentration of 60 × 10^6^, 40 × 10^6^, or 20 × 10^6^ cells/ml. Cells were then mixed with an equal volume of alginate solution (2 or 4% w/v in sterile RO H_2_O) resulting in final cell concentrations of 30, 20, and 10 × 10^6^ cells/ml in 1 or 2% alginate. The alginate/cell mixtures were extruded into a coagulation bath using a high precision KD Scientific KDS-410 constant volume syringe pump at a rate of 0.03 ml min^−1^. A 30G EFD Nordson A rotating drum was operated at a fixed velocity of 2 cm s^−1^ for the collection of fibers following coagulation. The collected fibers were immediately transferred to a solution of 154 mM HEPES and 10 mM NaCl to avoid drying and cell damage.

For PLA:PLGA fibers, the same apparatus was used with the modification that 20 wt % 75:25 PLA:PLGA (110,000 MW; 0.71 i.v.) suspended in chloroform was coagulated (and washed) in isopropanol baths. The spinning solution injection and fiber collection rates were held, respectively, to 1.8 ml/h and 8.5 m/min as described elsewhere (Razal et al., 2009). The resulting fibers were seeded with myoblasts 48 h in Hams/F10 (TRACE) supplemented with 20% fetal bovine serum (Invitrogen), 2.5 ng ml^−1^ bFGF (PeproTech Asia), 2 mM L-glutamine (Invitrogen), 100 U ml^−1^ of penicillin (Invitrogen), and 100 mg ml^−1^ streptomycin (Invitrogen) before they were implanted into mouse muscle.

To populate the PLGA fibers with cells, MPCs were grown *ex vivo* on the PLGA fibers as described elsewhere (Razal et al., 2009) for 7 days at which point they were detached from the substrate for implantation into recipient *mdx* and *wt* mouse muscle.

### Analysis of Biosynthetic Trojan Horse Fiber Muscle Tissue Constructs (TH-fMTCs)

**Figure 2:**
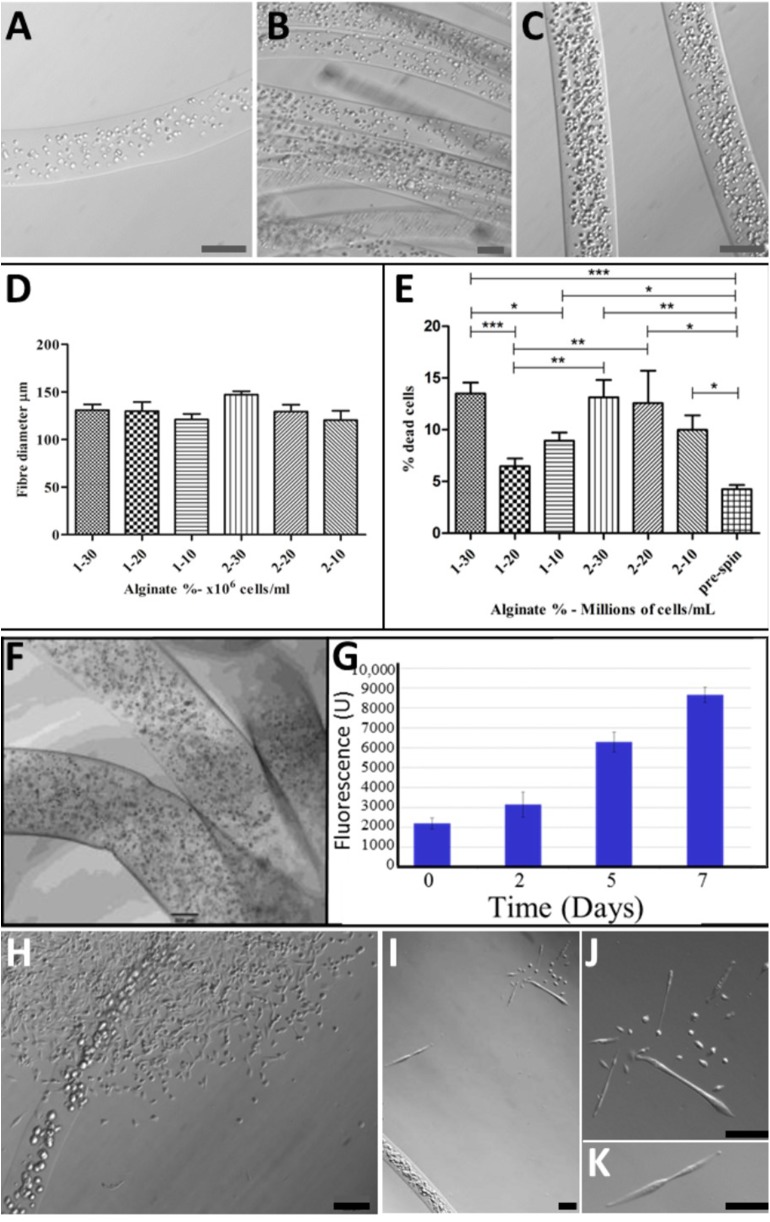
Optimization of BioSynthetic Alginate/Myoblast Trojan Horse Constructs for Implantation: Alg Fibers with: **(A)** 1 × 10^7^ cells/ml; **(B)** 2 × 10^7^ Cells/ml; **(C)** 3 × 10^7^ Cells/ml; **(D)** Controlled and consistent fiber diameter regardless of cell numbers or alginate concentration was achieved, with no statistical differences evident between syntheses (*t*-test). **(E)** Cell Death (%Dead Cells) was evaluated in 1 and 2% alginate fibers loaded with 1, 2, and 3 × 10^7^ Cells/ml to establish optimal alginate/cell combination as evident by post-spun cell viability. A maximum of ~15% of cells died as a result of wet-spinning in 1 and 2% alginate loaded with 3 × 10^7^ Cells/ml and in biosynthetic fiber constructs of 2% alginate loaded with 2 × 10^7^ Cells/ml. This evaluation showed that 1% Alg/2 × 10^7^ Cells/ml resulted in the least numbers of cells entering programmed cell death as a result of the wet-spinning process. Statistical-significance markers for student *t* statistics shown are as follows: *p < 0.05; **p < 0.01; ***p < 0.005. **(F)** Cells disperse inside the BioSynthetic 1% Alginate/2 × 10^7^ myoblasts/ml Trojan Horse constructs *in vitro*, where they **(G)** Proliferate freely until they **(H)** Migrate from the constructs into the surrounding environment and **(I–K)** Differentiate normally according to biological cues within the environment. This biological behavior *in vitro* models the cell delivery dynamic expected when the cell-loaded alginate-based Trojan Horse constructs are implanted into muscle. Unless otherwise specified, size calibration bars are 100 μm. Optimization of PLGA/Cell fibers was limited to seeding cells on fibers at optimal *ex vivo* cell plating density immediately prior to implantation. Unless otherwise specified, size calibration bars are 100 μm.

For viability and characterization, cell-bearing alginate biofibres were collected into HEPES containing 1 μM calcein AM in DMSO and 1 μg ml^−1^ of propidium iodide (PI). An Olympus IX70 fluorescence microscope was used at 10× magnification to visualize fibers with varying cell densities (Figures 2A–C) and to establish optimal Alginate concentration (Figure 2D) and cell density (Figure 2E) for the alginate-based TH-fMTCs.

For quantification of DNA content within the fibers, a PicoGreen assay was used. For each subgroup, five sets of twenty 10 mm fibers were added to a microfuge tube containing 100 μL 0.1% wt v^−1^ Triton-X in PBS solution. For cryo-sectioning, samples of fibers were embedded into Tissue-Tek Optimal Cutting Temperature Compound and placed into a slurry of dry ice and ethanol. These were stored at −70°C before being sectioned using a LEICA CM1900 Rapid Sectioning Cryostat.

In all analyses, four independent synthesis experiments were evaluated to confirm/otherwise the repeatability of the fabrication process. Finally, several fibers were collected into both proliferation and differentiation media for visualization of cell placement within the fibers and to establish biomimetic support of key pro-myoregenerative behavior (proliferation, differentiation and migratory capabilities) of the cells encapsulated within the alginate fMTCs (Figures 2G–K).

### Surgical Implantation of Trojan Horse Fibers and Cell Bolus Into *mdx* and *wt* Muscle

C57BL/10ScSn/Arc (wild-type; *wt*) and C57BL/10ScSn-Dmd^mdx^/Arc (X-Linked Muscular Dystrophy) male mice, 8–9 weeks of age were obtained from Animal Resources Center, WA, exposed to a 12 h day/night cycle and fed *ad-libitum* until they were required for surgery. All animal handling was performed according to the St. Vincent's Hospital Animal Ethics Committee protocol 013/11, in accordance with the Australian Code of Practice for the Care of Animals for Scientific Purposes (NHMRC).

**Figure 3:**
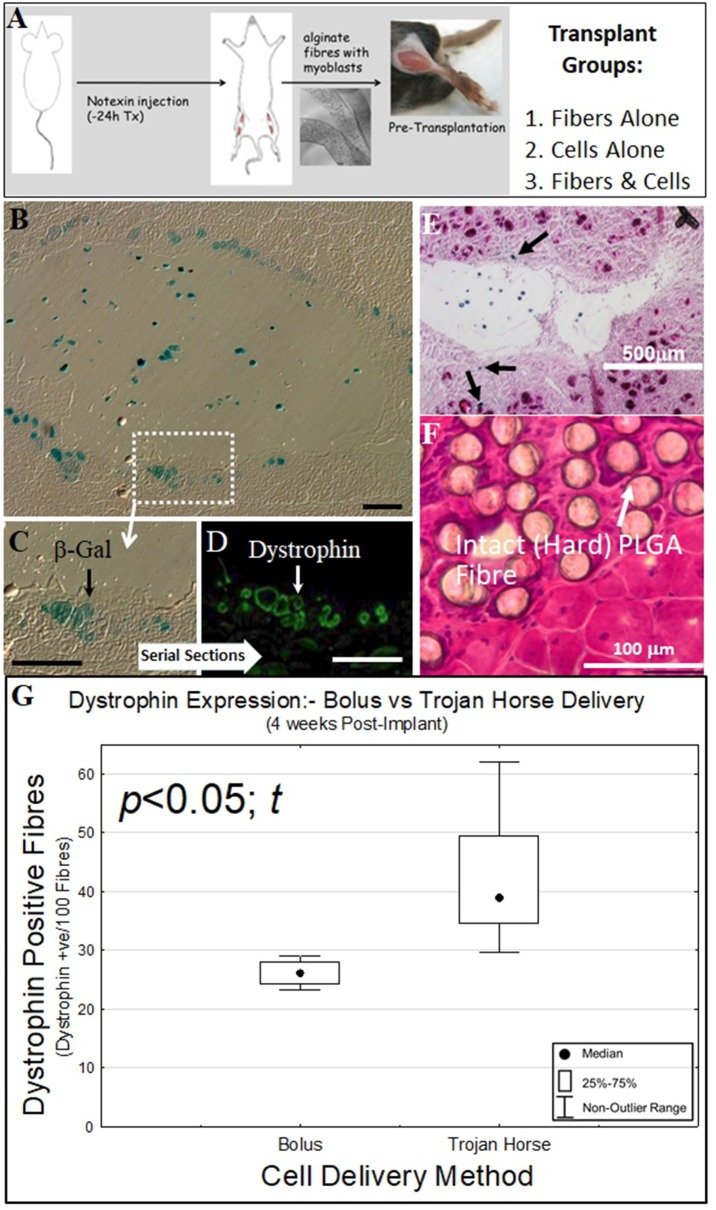
Biosynthetic PLGA and alginate fiber and cell bolus implantation into dystrophic and damaged mouse muscle. **(A)** Experimental schematic for implantation of biosynthetic cell/fiber constructs containing dystrophin and β-Galactosidase-expressing C57BL10/β-Gal mouse myoblasts into *mdx* and C57BL10J mouse muscle. **(B)** Biosynthetic 1% Alginate Trojan Horse Fibers loaded with β-Gal transgenic C57BL10/β-Gal myoblasts (3 × 10^6^ c/ml) were transplanted into dystrophic C57BL10-*mdx* mouse (muscular dystrophy) muscle showed that donor cells that migrated into the surrounding muscle expressed both (blue) β-Gal **(C)** As well as (green) dystrophin **(D)** In the dystrophic *mdx* host muscle. **(E)** This behavior also occurred in non-dystrophic muscle, with β-Gal-positive cells evident within the Trojan Horse construct and in fibers (Eosin/β-Gal-stained) of the surrounding muscle. **(F)** Myoblasts implanted on PLGA fibers did not survive and the PLGA fibers were still intact within the recipient muscle at 4 weeks post-implantation, with the PLGA fibers remaining hard (H&E stain). This was evident even at 3 months post implant. **(G)** Alginate Trojan Horse constructs yielded demonstrably more effective re-modeling of recipient dystrophic muscle with dystrophin-expressing cells (*p* < 0.05; *t*) than either cell bolus or cells implanted on PLGA fibers. PLGA fibers implanted with cells attached yielded no donor cells engrafted into the host muscle tissue at this timepoint (data not shown) and were discounted from further consideration as a cell delivery vehicle. Unless otherwise specified, size calibration bars are 100 μm.

All surgical procedures were performed under anesthesia using inhaled isofluorane (1% v:v, 1 L min^−1^ O_2_). The hind leg fur was removed by shaving and 10 μl of 5 μg/ml notexin (Sigma-Adlrich) was injected into the gastrocnemius muscle of both legs. The following day the gastrocnemius muscles were exposed by an incision through the skin. A 3–4 mm deep, 5 mm long incision was made in the muscle parallel to the muscle fibers with a scalpel blade. The incision was teased open with tweezers and 5 mm long cell-laden alginate or PLA:PLGA fibers (~5 × 10^5^ cells) were placed inside (Figure 3A). The muscle was closed by suturing the muscle fascia with a 6/0 silk suture (Dynek) and the skin closed with 9 mm wound clips (Becton Dickinson). All mice received subcutaneous injection of (Carprofen, 5 mg kg^−1^) analgesic post-surgery.

Cell Bolus groups were injected with 1 × 10^6^ cells suspended in mouse-tonicity saline after the notexin treatment.

At selected post implantation analysis times, mice were euthanized by cervical dislocation, and gastrocnemius muscles removed. The muscles were snap frozen in liquid nitrogen-cooled 2-methylbutane (Sigma-Aldrich) and stored at −70°C prior to analysis.

Muscles were cryosectioned by cutting 10 μm serial cross-sections through the entire length of the specimen to find localization of donor (βGal^+ve^) cells.

### Beta-Galactosidase (βGal) Staining

Cryosections were equilibrated to room temperature and fixed in 0.5% gluteraldehyde in PBS (Sigma-Aldrich) for 5 min at room temperature. Sections were washed in three changes of PBS and staining solution (2.5 mM K_3_Fe(CN)_6_), 2.5 mM K_4_Fe(CN)_6_.3H_2_0, 2 mM MgCl_2_, 1 mg ml^−1^ X-Gal, 1× PBS pH 7.4) was applied. Sections were incubated overnight at 37°C in a humidified chamber. At completion of staining sections were washed in two changes of distilled water and mounted in aqueous mounting medium. In some cases, sections were back-stained with Haematoxyllin and Eosin as described below (Figures 3B,C,E,F). The specimens were visualized using an Olympus IX-70 fluorescent microscope, and images were taken using Spot Advanced 4.0.9 software.

### Dystrophin Immunofluorescence

Cryosections were equilibrated to room temperature and rinsed in phosphate buffered saline (PBS, pH 7.4). Sections were blocked in 10% v/v donkey serum in PBS for 60 min, then incubated with either rabbit anti-dystrophin antibody (Santa Cruz) at 1:100 dilution in blocking solution for 60 min at 37°C, followed by two washes with PBS. Secondary donkey anti-rabbit Alexa Fluor 488 (Molecular Probes) diluted 1:2,000 in blocking solution was incubated for 60 min at 37°C. After two washes in PBS, nuclei were stained with 1 μg ml^−1^ DAPI (4,6-diamidino-2-phenylindole dihydrochloride) (Sigma–Aldrich) for 5 min and the specimens mounted in Fluorescence Mounting Media (DAKO). The specimens were visualized using an Olympus IX-70 fluorescent microscope, and images were taken using Spot Advanced 4.0.9 software (Figure 3D). Dystrophin positive fibers were counted for PLGA (*n* = 5) and Alginate (*n* = 5) Trojan Horse constructs as well as for cells injected as a cell Bolus (*n* = 4) as shown in Figure 3G.

### Hematoxylin and Eosin Staining

Cryosections were equilibrated to room temperature and fixed in 10% Formalin (Sigma-Aldrich) for 5 min at room temperature. Sections were washed in tap water, stained with Gills' Hematoxylin (Sigma-Aldrich) for 30 s, rinsed in 30 immersions into tap water (water changes every 10 immersions), stained in Alcoholic Eosin Y (Sigma-Aldrich) with 2 immersions, rinsed in 30 immersions of tap water (3 water changes), dehydrated in three changes of 100% ethanol and cleared in xylene. Sections were mounted in DPX. The specimens were visualized using an Olympus IX-70 fluorescent microscope, and images were taken using Spot Advanced 4.0.9 software.

## Results

The fabrication process (Figures 1A,B) of PLGA (Figures 1D,E) and alginate (Figure 1F) Trojan Horse fibers containing myoblasts and subsequent biological viability of these fibers were optimized *in vitro* by analysis of process parameters: PLGA fibers were maximally over-grown with myoblasts immediately prior to further studies, whilst evaluation of alginate concentration and cell-loading parameters reproducibly yielded 1% and 2% alginate fibers containing large numbers of cells. PLGA/myoblast constructs required no further evaluation/optimization. Cell numbers in alginate Trojan Horse Fiber Mus were controlled by material feed rate and handling methods, reliably yielding fibers that contained between 1 and 3 × 10^7^ cells (Figures 2A–C). The fiber diameter was able to be controlled likewise via material feed rate, to between ~130 and 150 μm, with an average diameter of 125 μm regardless of incorporated cell loading density or alginate (w/v) concentration (Figure 2D).

Cell loading density was evaluated by extraction of DNA from the synthetic bio-fibers and quantification using PicoGreen assay for nucleic acids and was shown to increase accordingly with cell numbers suspended within the originating alginate solution. Of the six configurations evaluated, cell viability (based on PI infiltration assay) with respect to cell loading and percentage (w/v) Alg, was highest in fiber configurations of 1% alginate containing 20 million cells/ml (96 ± 1%) (Figure 2E). These fibers were taken as optimal synthetic bio-fibers for subsequent experiments. Fibers produced using 2% alginate as a matrix material, while displaying similar fiber diameters, demonstrated a lower cell viability and consistency compared to correspondingly cell-loaded 1% alginate fibers (Figure 2E).

The synthetic Trojan Horse bio-fibers were modeled in the first instance on native muscle fiber/myoblast structures observed in early primary culture stages (Figure 1C) and compared to PLGA/myoblast fiber constructs (Figures 1D,E) as possible structures for regenerative cell delivery into muscle. Histological evaluation of these cell-laden fibers showed that the myoblasts were evenly distributed longitudinally along and cross-dimensionally across the diameter of the alginate fibers (Figure 1F), whilst the PLA:PLGA fibers (Figure 1D) showed distinct similarity to native fiber explants (Figure 1C).

To see if these constructs could be used to deliver functional cells to muscle tissues, optimized biosynthetic fiber constructs containing transgenic C57BL10J-βGal mouse myoblasts in alginate or on PLA:PLGA fibers were prepared for implantation into mouse muscle (Figure 3A): Synthetic (alginate) bio-fibers of ~500 μm diameter were wet-spun using 1% alginate and myoblasts at a density of 3 × 10^5^ cells/ml derived from non-dystrophic, β-Gal-expressing C57BL10J-ScSn^βGal^ mice (Figure 2F). Well below the 20 × 10^6^ cells/ml viability threshold for 1% alginate fibers (Figure 2E), this low cell density facilitated reliable inclusion of ~3,000 cells per 5 mm fiber implant for (quantitative) tracking of post-transplantation cell fate within the recipient muscle tissue. The use of the C57BL10J-βGal mouse myoblasts provided an allogeneic donor cell source that facilitated post-implantation tracking of implanted cells' localization via the βGal transgene whilst the larger diameter facilitated manageability of handling during surgery.

PLA:PLGA fibers of ~130 μm diameter were likewise seeded with C57BL10J-βGal mouse myoblasts as previously described, for implantation into the recipient *mdx* mouse muscle.

Prior to implantation, the synthetic alginate bio-fibers' cell-loading and viability were confirmed by microscopy (Figure 2E) and the integrated cells' ability to proliferate within the fibers was confirmed over 7 days' culture *in vitro*. This established that myoblasts embedded in the alginate fibers could be expanded to at least four-fold the original cell numbers over 7 days (Figure 2G) to attain required cell numbers if necessary. The myoblasts displayed the ability to migrate from the alginate fibers and to proliferate (Figure 2H) and differentiate (Figures 2I–K) within the *in vitro* culture environment, a precursor indication of the fibers constructs' expected biological behavior when implanted into muscle. Likewise for the PLA:PLGA fibers (Figure 1D). Cells seeded on PLA:PLGA fibers likewise retained proliferative and migration abilities *in vitro* (data not shown).

The bio-synthetic constructs (5 mm in length) were surgically implanted into notexin myo-ablated gastrocnemius muscles of C57BL10J ScSn (*wt*) and C57BL10J ScSn^*DMD*^ (*mdx*) mice. These fibers were left in the recipient mouse muscles for 1 and 2 weeks, respectively, after which the mice were euthanized and the tissues harvested for analysis of (i) cell survival within the constructs, (ii) cell migration from the constructs into the host muscle, and (iii) ability to remodel the host muscle with regenerative muscle fibers derived from the donor cells.

Donor cells implanted in alginate Trojan Horse fMTCs into both *mdx* (Figure 3D) and C57BL10J non-dystrophic (Figure 3E) mouse muscles survived within the host tissue 4 and 2 2 and 1 week post implantation, respectively. In both dystrophic and non-dystrophic environments, donor cells were evident both within the boundary of the alginate fiber component in which they had been implanted, as well as within the recipient mouse muscle tissue. Serial sections of the biosynthetic fiber construct-implanted *mdx* muscle showed that expression of the βGal transgene from the non-dystrophic C57BL10J-ScSn^βGal^ derived donor myoblasts (Figures 3B,C) corresponded with dystrophin expression from the donor myoblasts within the implanted *mdx* muscle (Figure 3D). In addition, there were clear signs that the alginate vehicle used to deliver the cells into the muscle environment had begun to undergo degradation even after 1 week, in stark contrast to PLA:PLGA/myoblast fibers, which were clearly evident as intact in recipient muscle after 12 weeks without cell survival and with significant foreign body response (Figure 3F). These results suggest that *in vitro* myoblast proliferation in, and migration from a biosynthetic alginate/cell construct with subsequent differentiation to muscle fibers within the surrounding environment shown *in vitro* in Figures 2I–K translate to cell behavior from within such constructs when implanted into muscle tissue *in vivo*. On the other hand, the lack of cell engraftment into host *mdx* muscle by cells seeded on PLA:PLGA fMTCs indicates that this mode of delivery is unsuitable for delivery of cells to muscle tissues, whilst the alginate Trojan Horse-fMTC delivery method resulted in better engraftment of recipient muscle with donor cells that bolus injection of donor cell mass (Figure 3G).

## Discussion

Additive fabrication and assembly of functionalized fibers produced by wet-spinning into implantable three dimensional constructs presents attractive prospects for the field of regenerative medical bionics (Wallace et al., 2012). In particular, the incorporation of biologically active agents including growth factors (Quigley et al., 2013a,b) and living cells (Chung et al., 2013) within biocompatible and macroporous fibers that allow for mass transfer and cell migration, is expected to deliver improvements to cell and drug delivery platforms, particularly in the area of tissue engineering biotechnology (Hong et al., 2006; Lee et al., 2011).

This study investigated application of wet-spinning as an alternative (to 3D printing) additive bio-fabrication technology for generating three-dimensional cell-bearing (encapsulated and topically-seeded) biosynthetic fibers containing live muscle precursor cells (myoblasts) for remodeling damaged and dystrophic muscle.

Our previous studies evaluated a cell transplantation approach in which cells can be grown onto wet-spun fibers *ex vivo* for subsequent implantation into muscle tissue as biosynthetic fiber/cell structures. Razal et al. (2009) The cell-bearing fibers from earlier applications using poly(DL-lactic-co-glycolic acid) copolymer (PLA:PLGA) were shown here to be unsuitable muscle remodeling due to prolonged hardness and subsequent stimulation of fibrosis within the implanted muscle.

Subsequently, our long-standing use of sodium alginate (Alg) hydrogel cross-linked with calcium for delivery of myotrophic growth factors (Austin et al., 2000), identified Alg as a suitable material for muscle (i.e., soft tissue) engineering applications. Alg owes its suitability for such applications to its general physical compliance with soft tissue target properties and its permissive degradation profile that elicits minimal inflammatory response and facilitates passive diffusion of oxygen and mass transport (Qin, 2004; Berthiaume and Morgan, 2010).

In contrast to 3D bioprinting, which is somewhat limited to short, usually hatch-like structures, the structural linearity of wet-spun fibers mimics the linear characteristics of native muscle structure. Furthermore, wet spinning allows functional materials to be deposited, woven or knitted into defined structures with minimal disruption of incorporated biological factors' or cells' functional integrity (Chen et al., 2009). Perhaps most importantly, wet spinning affords precise control of placement and release of factors within and from the fibers, yielding more effective and reproducible fabrication of functionally defined devices and structures (De Ruijter et al., 2006; Mironov et al., 2009).

Further development of the wet-spun fiber/myoblast biosynthetic fiber system described here along these lines stands to deliver a highly defined regenerative cell construct for the repair and remodeling of damaged and diseased muscle that yields superior engraftment compared to bolus-injections of cell mass. In particular, refinement of the alginate component with biofactors (e.g., extracellular matrix and/or growth proteins) (Austin et al., 2000; Lee and Mooney, 2012) and possible pre-conditioning of the cells to specific defined sub-phenotypes will potentiate the value of these biosynthetic constructs for muscle engineering and repair.

**Figure 4:**
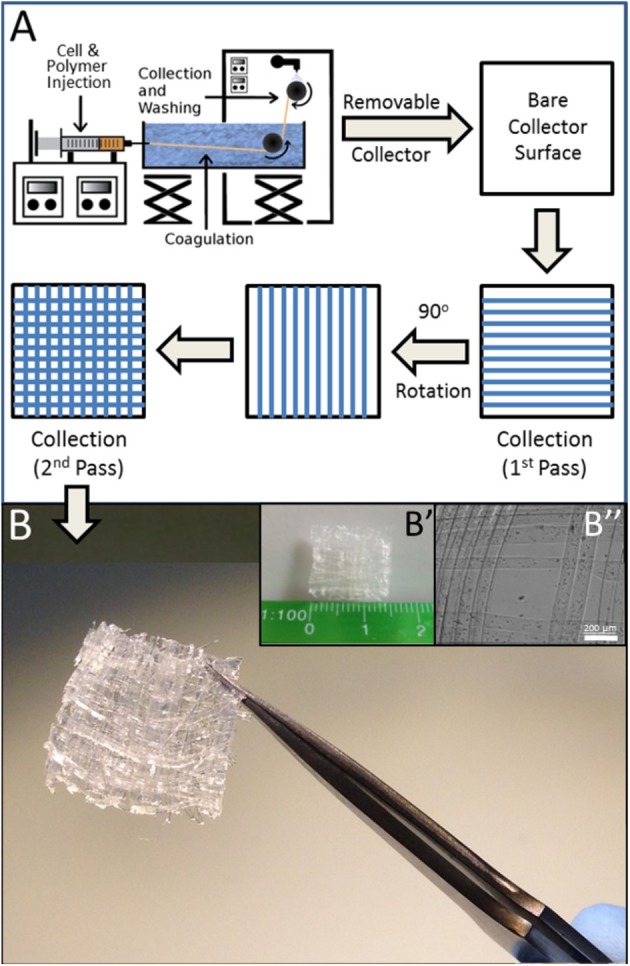
3D Matrix of BioSynthetic (Alg/Myoblast) Fibers. **(A)** Line schematic of layer by layer (LbL) deposition of fibers to form a 3D cell-containing mat. The process can be repeated additively with 90° rotation of each subsequent deposition prior to collection until the required number of layers are attained. Linearity of biosynthetic alginate/myoblast fibers can be maintained by alternating layers of just alginate (no cells) in between layers of alginate/myoblast fibers. **(B)** A 1 cm × 1 cm × 0.625 mm 3D 5-layer matrix scaffold of (1% alginate/2 × 10^7^ per ml myoblast) fibers prepared by LbL fiber deposition as described in **(A)**. (B′) The size of the entire 3D mesh scaffold shown against a millimeter scale. (B″) High resolution image of the mesh muscle tissue constructs, with cells located within the individual fibers.

A further point of refinement that better facilitates this system's application to tissue engineering is its translation to a three-dimensional format. Toward this end, the linear collection system outlined in Figure 1A was modified to incorporate a layer by layer approach in which fiber collection was periodically discontinued and resumed after a 90° adjustment of the collection axis (Figure 4A). In this manner, a 5-layer mat of wet-spun fibers containing cells was readily obtained (Figure 4B), demonstrative of further applicability of wet spinning to the construction of 3D myo-regenerative implants for applications of volumetric muscle loss.

Collectively, the wet spinning of cells into biocompatible fibers and subsequent formation of 3D structures from these fibers as described in this study provides a new avenue by which 3D muscle microtissues can be engineered *ex-vivo* for *in vitro* experimentation modeling or for re-engineering injured or diseased muscle. This layer by layer wet-spinning approach mitigates some of the issues such as exposing cells to excessive nozzle pressures evident in formation of such structures using other additive fabrication technologies such as 3D bio-printing.

## Conclusion

This work communicates the successful wet spinning of biosynthetic alginate/myoblast Trojan Horse fiber muscle tissue constructs (TH-fMTCs) composed of 1% alginate fibers containing primary myoblasts at high cell concentrations. The cells remained highly viable post-fabrication and it was determined that the most efficient subgroup consisted of a 1% wt/v alginate matrix incorporating a cell concentration of 2 × 10^7^ cells ml^−1^. The myoblasts embedded within the alginate fibers were shown to be proliferative *in vitro* and to migrate from the fibers into the surrounding environment, where they were able to undergo appropriate differentiation into myotubes. These regenerative biological behaviors were recapitulated *in vivo* by biosynthetic alginate/myoblast fiber constructs containing primary allogeneic C57BL10J-ScSn^βGal^ myoblasts carrying a βGal reporter transgene, implanted into myo-ablated non-dystrophic C57BL10J ScSn (*wt*) and dystrophic C57BL10J ScSn^*DMD*^ (*mdx*) mice. In these experiments, donor cell-derived βGal reporter transgene expression detected in muscle fibers outside of the alginate vehicle both in *mdx* and *wt* mouse muscle, confirmed that some of the donor myoblasts migrated from the biosynthetic fiber constructs, incorporated into the surrounding muscle tissue network and formed donor-derived muscle fibers. Importantly, co-expression of dystrophin (denoting functional, fully differentiated non-dystrophic muscle fibers normally absent in *mdx* muscle) with βGal in serial sections of the implanted *mdx* muscle highlighted that the biosynthetic alginate/myoblast constructs could remodel dystrophin-negative *mdx* muscle tissue with dystrophin-producing fibers.

In considering the material/cell configuration to be used for remodeling muscle tissue, the biosynthetic alginate/myoblast system reported here resulted in more robust regenerative results than did myoblasts attached to PLA:PLGA fibers, which left intact PLA:PLGA fibers devoid of donor myoblasts even 3 months after implantation. Likewise, the alginate TH-fMTC configuration yielded better engraftment of donor cells than did bolus-injected MPCs. These results suggest that 3D “Trojan Horse” scaffolds composed of biosynthetic soft gel alginate/myoblast fibers present an attractive method by which to promote remodeling of diseased and/or damaged muscle.

## Data Availability Statement

The datasets generated for this study are available on request to corresponding author RK.

## Ethics Statement

This study was approved by St. Vincent's Hospital Research Animal Ethics Committee Protocol 86/06.

## Author Contributions

AQ, RC, TM, MK, and JR performed the experiments. JF, JC, SM, GW, and RK supervised bench experimentation and planned the study. All authors provided intellectual input and collaboratively wrote the manuscript. RK and GW conceived and coordinated the study.

### Conflict of Interest

The authors declare that the research was conducted in the absence of any commercial or financial relationships that could be construed as a potential conflict of interest.

## Dedication

This work is dedicated to Professors Lawrie Austin and Leon Kane-Maguire, who would have greatly enjoyed working together and whose collective vision led to this work.
